# Calculation and Analysis of Key Parameters of Underwater Optical Imaging System

**DOI:** 10.3390/s24051537

**Published:** 2024-02-28

**Authors:** Guangpeng Zhou, Ying Liu, Boshi Dang, Chen Yu, Linhan Li, Jie Du, Junlin Ma, Xinyue Liu

**Affiliations:** 1 Changchun Institute of Optics, Fine Mechanics and Physics, Chinese Academy of Sciences (CIOMP), Changchun 130033, China; zhouguangpeng19@mails.ucas.ac.cn (G.Z.); dangboshi@163.com (B.D.); yuchen21@mails.ucas.ac.cn (C.Y.); lilinhan22@mails.ucas.ac.cn (L.L.); dj_ciomp@163.com (J.D.); michael8446@126.com (J.M.); liuxinyue@ciomp.ac.cn (X.L.); 2 University of Chinese Academy of Sciences, Beijing 100049, China

**Keywords:** underwater optical imaging system, optical window, focal length, depth of field

## Abstract

When photographing objects underwater, it is important to utilize an optical window to isolate the imaging device from the water. The properties of the entire imaging system will change, and the imaging quality will decrease due to the refraction impact of the water and the window. The theoretical calculation method for air imaging is no longer relevant in this context. To analyze the unique rule, this research derives the formulas for key parameters of underwater imaging systems under paraxial circumstances. First, the optical window is modeled, then the formula for the optical window’s focal length in the underwater environment is derived, and the change rule for the focal length of various window forms underwater is condensed. For the ideal imaging system using a domed optical window, the equivalent two-optical group model of the imaging system is established, and the formula for calculating the focal length, working distance, and depth of field of the underwater imaging system is derived through paraxial ray tracing. The accuracy of the formula is verified through the comparative analysis of the formula calculation results and the Zemax modeling simulation results. It provides an important theoretical basis for the in-depth study of underwater imaging technology.

## 1. Introduction

Underwater imaging technology is the basis for understanding and developing the ocean, optical imaging is currently the primary technological way of underwater imaging [1,2], which is widely used in underwater exploration. Underwater optical imaging still faces considerable challenges [3]. In comparison to air, water is a more complex medium. First of all, the absorption of light energy by water is significantly greater than air, limiting the imaging distance. Selective absorption of water also causes color distortion in the image [4,5]. The non-uniform composition of the water, as well as the presence of various impurities that scatter the light, causes the detector to receive a non-uniform distribution of light energy, degrading the image quality and reducing the contrast [6]. Active lighting must also be taken into account in deep-sea environments, where natural light is almost entirely lost. Additionally, equipment must be designed with a “hard shell” to survive the pressures of deep water [7,8].

Underwater optical imaging equipment often consists of a camera with a sealed, pressure-resistant housing. The housings have transparent windows in the form of a flat or dome to allow light to pass through. These housings safeguard the imaging devices, but they also create new problems. Most windows are made of hard, transparent plastic, glass, sapphire, or ceramics, which are media with a refractive index higher than that of water. Light passes through the water-window interface before entering the imaging device. According to the law of refraction, if the light is not incident perpendicular to the water-window interface, then the light will be refracted at the interface, and the refraction at the interface will change the original propagation path of the light. These light rays that deviate from their original paths tend to reduce the field of view of the imaging device when they pass through a flat window [9], and when they pass through a hemispherical dome window, they create new aberrations in the entire imaging system that can damage the quality of the image [10]. This means that even if these optics are well designed and calibrated in air, they do not meet the requirements of the initial design when working in an underwater environment due to the combined effect of the flat plate or dome window and the water.

Because the object-space medium changes, more refraction occurs before the light enters the imaging system, which causes the separation of the main and nodal planes of the imaging system; G. T. McNeil et al. redefined and standardized underwater photographic optics terminology by proposing that the base point of an underwater lens system translates [11]. In previous studies of the use of optical imaging theory, the case where the spatial medium of the object and image is all air is well established. The study of underwater optical imaging typically falls within the category of multimedia imaging, where multimedia denotes the passage of light through different media before it is collected by the imaging device: the water where the target is located, the window material, and the air in the space where the imaging device is located. When J.-M. Lavest et al. examined sensor calibration in various media, they concluded that the optical laws must be reconsidered when using an underwater camera, extending the pinhole model in different media to a nodal model, and concluded that the focal length of an underwater imaging system is 1.33 times greater than in air [12]. According to P. Agrafiotis et al., the amount of water and air in the overall distance between the camera and the object affects the effective focal length of the camera in dual-media imaging [13]. However, both of these laws only apply to systems that use flat windows. F. Menna et al. also tested a commercial underwater camera in a pool and used photogrammetric techniques to describe the optical phenomena of flat windows and dome windows [14], but they lacked precise calculations. G. Jiang et al. modeled and analyzed the imaging process of an underwater plenoptic camera and proposed an optimization method for an underwater plenoptic camera [15]. However, this method is not generalizable and is only relevant to plenoptic cameras using microlens arrays. In conclusion, while existing research offers several techniques for underwater camera calibration optimization, the lack of direct quantitative calculations and analysis of the effects of water and optical windows (especially dome windows) on imaging equipment, and the lack of a general calculation formula for the properties of underwater imaging system limit the development of underwater imaging technology. Therefore, it is necessary to make accurate calculations on the performance changes of imaging equipment underwater for the special characteristics of underwater imaging.

In this study, we first model the window individually. Subsequently, we derive a formula to determine the focal length of the window within the underwater environment, specifically in the image space, and we explore how the focal length of the window varies when subjected to two distinct shapes of the water-window interface. Secondly, in the case of the dome window, we conducted calculations to assess how the image-square focal length changes in response to variations in the refractive index of water. Additionally, we pinpointed specific shape features that exhibit reduced sensitivity to water-induced effects. To precisely compute the imaging properties of an imaging system featuring a dome window, we devised a simplified geometric model. This model accounts for alterations in the imaging system’s cardinal points and establishes geometric relationships, allowing us to formulate equations for both the total focal length of the system and the underwater front working distance. Based on the reversibility of light, we establish a relationship between the front working distance and the refractive index of the object space. Additionally, we develop a comprehensive depth-of-field formula specifically designed for underwater environments, enhancing the precision of performance evaluations for underwater imaging systems.

## 2. Underwater Optical Window Imaging Properties

Imaging equipment in the underwater environment, by waterproof shell and optical window to isolate the water. The window functions as a thick lens in the system due to the refractive index of the window material and its thickness to resist water pressure. As a result, underwater imaging has an extra optical element in the construction of the device, unlike aerial imaging technologies that directly picture the target. The target object located in the water first produces a virtual image in front of the window lens, which is also the virtual object of the imaging device, and then the imaging device focuses on it. The virtual object changes the object distance when the imaging device is working. If the position of the virtual object exceeds the focus range of the imaging device, the image will be blurred. More optical structures or other methods need to be added to meet the imaging requirements. According to the definition of applied optics, the position of the virtual image formed by the window lens is closely related to its imaging properties, such as focal length and cardinal point position, so we first analyze the focal length of the window lens and the cardinal points and other imaging properties.

**Figure 1:**
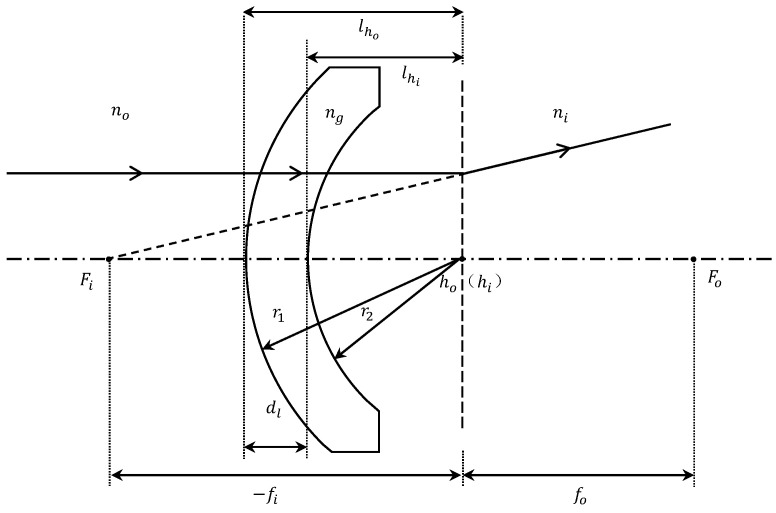
Model of a domed window, with the parameters of the window as a form of lens and the focal points and principal points under the definition of applied optics labeled in the figure.

As shown in Figure 1, taking the dome window as an example, the calculation of the focal length and cardinal point of the window follows the definition of geometrical optics, where the focal length represents the distance from the principal point to the focal point. In addition, all our calculations below follow the sign conventions in applied optics: (1) light traveling to the right is positive; (2) the axis segment measured from the lens reference point to the right is positive, while to the left is negative; (3) the vertical axis line segment above the optical axis is considered positive, whereas below is negative.

The working environment of the window is special. The image space is an air medium, the object space is water with a larger refractive index, and the refractive index of water in the actual working environment is not yet fixed [16]. In the case of different media in the object and image space, the focal length formula of the window lens can be varied from the thick lens focal length formula [17]:

(1) $$-\frac{n_{o}}{f_{ol}}=\frac{n_{i}}{f_{il}}=\frac{n_{g}-n_{o}}{r_{1}}\left(1-\frac{n_{i}-n_{g}}{r_{2}}\frac{d_{l}}{n_{g}}\right)+\frac{n_{i}-n_{g}}{r_{2}}$$

where $f_{ol}$ and $f_{il}$ are the object and image-space effective focal length of the window lens; $r_{1}$ and $r_{2}$ are the front and rear curvature radius of the window; $n_{o}$ and $n_{i}$ are the refractive index of the object and image space; $n_{g}$ is the refractive index of the window lens; $d_{l}$ is the axial thickness of the window lens.

The left side of Equation (1) is the ratio of the refractive index to the focal length, also known as optical power $\varphi_{l}$, which represents the ability of the lens to converge or diverge light rays:

(2) $$\varphi_{l}=\frac{n_{g}-n_{o}}{r_{1}}\left(1-\frac{n_{i}-n_{g}}{r_{2}}\frac{d_{l}}{n_{g}}\right)+\frac{n_{i}-n_{g}}{r_{2}}$$

Substituting Equation (2) into Equation (1) so that the focal lengths in the image and object space of the window lens can be expressed as follows:

(3) $$\left\{ \begin{array}{l} f_{il}=\frac{n_{i}}{\varphi_{l}}\\ f_{ol}=-\frac{n_{o}}{\varphi_{l}} \end{array}\right.$$

From Equations (2) and (3) above, the focal length is a quantity that is influenced by the structural properties of the window lens (radius of curvature, thickness, refractive index of the material) and the refractive index of the object space. In underwater imaging, the refractive index of the object space ($n_{o}$) is the main influencing factor of the change in focal length. In the underwater environment, the differing refractive index of the media on both sides of the window ($n_{o}$,$n_{i}$) leads to the window lens having two focal lengths, $f_{ol}$ in the object space and $f_{il}$ in the image space. The magnitude ratio of these focal lengths corresponds precisely to the ratio of the refractive index of the respective media.

Furthermore, the configuration of the water–window interface ($r_{1}$) plays a pivotal role in influencing how the refractive index of the object space ($n_{o}$) impacts the focal length of the window lens.

1. When $r_{1}=\infty$, the window interface in contact with the water adopts a flat configuration. Equation (2) becomes:
  (4) $$\varphi_{l}=\frac{n_{i}-n_{g}}{r_{2}}$$

In Equation (4), the optical power denoted as $\varphi_{l}$ is determined solely by the final term, establishing a constant value that remains unaffected by variations in the refractive index of the object space. Then, substituting Equation (4) into Equation (3) provides the following:

(5) $$\left\{ \begin{array}{l} f_{il}=\frac{n_{i}}{\varphi_{l}}=\frac{n_{i}r_{2}}{n_{i}-n_{g}}\\ f_{ol}=-\frac{n_{o}}{\varphi_{l}}=-\frac{n_{o}r_{2}}{n_{i}-n_{g}} \end{array}\right.$$

As shown in Equation (5), the focal length in the image space of the window ($f_{il}$) at $r_{1}=\infty$ remains unaffected by the refractive index of the object space ($n_{o}$), maintaining identical values in both air and water. In contrast, the focal length in the object space ($f_{ol}$) is directly proportional to $n_{o}$, and when underwater, $f_{ol}$ becomes $n_{o}$ times larger than in air.

**Figure 2:**
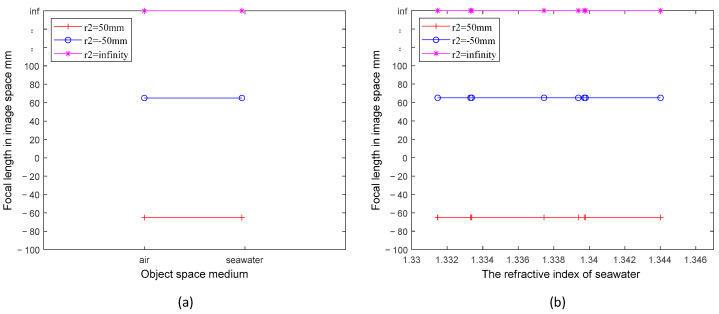
(**a**) Comparison of the focal length values of three different $r_{2}$ of the front flat window in air and seawater, respectively, with the refractive index of seawater taking the value of 1.339; (**b**) variation of the focal length values of three different $r_{2}$ of the front flat window in seawater with different refractive indices.

Consider three distinct structural window types: plano-concave, plano-convex, and flat. Each adheres to the condition $r_{1}=\infty$, with rear curvature radius ($r_{2}$) at 50 mm, −50 mm, and infinity, correspondingly, all constructed from sapphire. The image-space focal length variation curves of the three windows in media with different refractive indices are obtained by the calculation of Equation (5). In Figure 2, the depicted lines remain linear regardless of the medium transition from air to seawater or within seawater at different refractive indices. The focal length of the window’s image space for the three structures remains consistently fixed, determined by their individual structural properties, specifically the size of $r_{2}$, and the materials employed. The property of having $r_{1}=\infty$, termed as front-flat, bestows an advantage upon the window: the image-space focal length remains unaffected by alterations in the refractive index of the object space medium. While operating underwater, this front-flat window diminishes the impact of medium changes on the camera. However, it regrettably reduces the camera’s field of view [9].

2. In cases where $r_{1}\neq \infty$, the window-water interface takes on a curved configuration, leading to increased complexity. This curvature introduces the possibility of an angle forming between the incident infinity ray and the surface’s normal vector. As stipulated by the law of refraction, the angle of outgoing radiation hinges upon both the refractive index of the object space and the angle of incidence. Consequently, the location of the focal point in the dome-shaped window lens undergoes changes contingent on variations in the refractive index of the object space and the configuration of the interface, $r_{1}$ and $r_{2}$.

Typically, in underwater applications, curved windows are predominantly of the domed variety, featuring front and rear surfaces that conform to concentric spheres. Specifically, the thickness is $d_{l}=r_{1}-r_{2}$. The concentric configuration offers the dual benefit of withstanding pressure while ensuring a consistent field of view size [10]. Generally, air serves as the medium between the dome window and the imaging device, thus determining the focal length of the window lens as follows:

(6) $$-\frac{n_{o}}{f_{ol}}=\frac{1}{f_{il}}=\frac{n_{g}-n_{o}}{r_{1}}\left(1-\frac{1-n_{g}}{r_{2}}\frac{d_{l}}{n_{g}}\right)+\frac{1-n_{g}}{r_{2}}$$

The complexity of the lens focal length is evident as it depends on both $n_{o}$ and $r_{1}$ and $r_{2}$. There is no longer a fixed ratio law for underwater focal length and air either. Parameters for underwater operation cannot be obtained using land-based laboratory calibrations. The window’s focal length value can be determined solely by employing Equation (6) once the refractive index of the water medium is known.

According to Equation (6), the alteration in window focal length primarily results from variations in $n_{o}$, while the extent of this change is collectively influenced by the surface curvatures $r_{1}$ and $r_{2}$. Using an actual window calculation as an example, we delve into examining the specific effects of $r_{1}$ and $r_{2}$.

**Figure 3:**
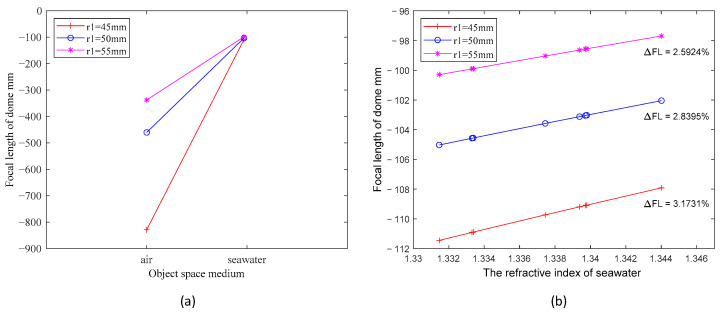
(**a**) Comparison of the focal length values of three dome windows with different $r_{1}$ in air and water, respectively, with the water refractive index taking the value of 1.339; (**b**) the variation of focal length values for dome windows with three different $r_{1}$ in seawater with varying refractive indices.

First, we take three windows whose materials are all sapphire, with consistent $r_{2}$ values of 40 mm. The thickness of underwater dome windows is generally within 5–15 mm, so the corresponding $r_{1}$ values are 45 mm, 50 mm, and 55 mm, respectively. The refractive index of the water is considered within the range of 1.33 to 1.36 [16,18]. In accordance with the predefined parameters and Equation (6), we computed the alteration in the focal length in the image space of the three windows when transitioning from air to water with a refractive index of 1.339. The outcomes are depicted in Figure 3a. Then, we calculated the focal length values in the image space for the three windows while varying the water’s refractive index within the range of 1.33 to 1.35. The findings are presented in Figure 3b. The negative focal length in the image space of the dome window indicates that the window acts as a negative lens. The negative sign of the focal length value represents the relative direction of the focus and the window, signifying that the image focus is situated to the left of the window. The variations in the curves depicted in both figures reveal that the refractive index of the object space ($n_{o}$) exerts the primary influence on the focal length alterations of the window. This effect is particularly pronounced as the refractive index undergoes a substantial shift from air to an aqueous medium, leading to a significant reduction in the window’s focal length. Likewise, the decrease in focal length observed in Figure 3b can be attributed to the elevation of the water’s refractive index. This phenomenon precisely corresponds to the negative lens effect of water [19]; it can be analogized to introducing a negative lens ahead of the window, causing light to diverge away from the optical axis. Importantly, the greater the refractive index of the water, the more pronounced the divergence effect, resulting in a commensurately diminished focal length of the window. The magnitude of the front curvature radius ($r_{1}$) of the window plays a pivotal role in defining the extent to which the refractive index of water affects the window’s focal length. This phenomenon is illustrated in Figure 3b, where the three curves of $r_{1}$ exhibit varying degrees of sensitivity to changes in the refractive index of water within the range of 1.330 to 1.345. For instance, when $r_{1}$ is 45 mm, the window’s focal length experiences a change of approximately 2.59%. However, as $r_{1}$ increases to 55 mm, the window’s focal length undergoes a more limited change, approximately 3.17%. This phenomenon becomes notably more pronounced when transitioning from air to an underwater environment, as illustrated in Figure 3a. A greater radius of curvature ($r_{1}$) results in reduced susceptibility to negative lensing effects and, consequently, minimized alterations in focal length.

**Figure 4:**
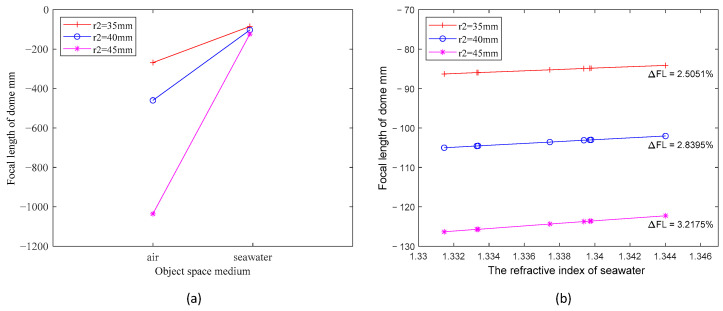
(**a**) Comparison of the focal length values of three dome windows with different $r_{2}$ in air and water, respectively, with the water refractive index taking the value of 1.339; (**b**) the variation of focal length values for dome windows with three different $r_{2}$ in seawater with varying refractive index.

Subsequently, we calculate windows with varying rear curvature radius ($r_{2}$). Again, we use the example of three windows, where $r_{1}$ values are 50 mm, and $r_{2}$ values are 35 mm, 40 mm, and 45 mm, respectively. The remaining parameters are kept consistent with those detailed in the preceding paragraph. Employing Equation (6), we compute and generate the two graphs displayed in Figure 4. Once more, the primary factor driving the curve variations is the refractive index $n_{o}$ within the object space. In Figure 4b, the three curves exhibit varying degrees of change within the seawater refractive index range of 1.330 to 1.335. Notably, the dome window with a 45 mm rear radius of curvature displays a shift in image-space focal length of approximately 3.22%, while the window with a 35 mm rear radius of curvature demonstrates a range of change limited to about 2.51%. The observation depicted in Figure 4b illustrates that a smaller radius of curvature $r_{2}$ corresponds to reduced exposure of the window to the negative lensing effect, resulting in a smaller change in focal length.

In underwater applications, two prevalent window types are flat windows and dome windows. The calculations presented above reveal that the focal length in the image space of the flat window remains unaltered in the underwater environment, in contrast to the imaging characteristics of the dome window, which exhibit greater susceptibility to variations in the refractive index of water. Although selecting a dome with appropriate shape characteristics can mitigate the impact of the water refractive index, this factor cannot be disregarded. Hence, to thoroughly assess the imaging of the dome window, more intricate calculations are necessary. In Section 3, we will construct a comprehensive computational model to investigate variations in the imaging of the dome window system.

## 3. Object-Image Relationships for Underwater Optical Imaging Systems

**Figure 5:**
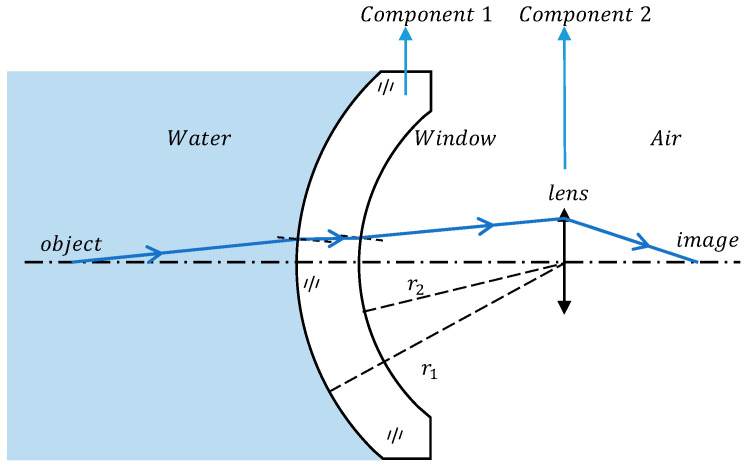
A computational model consisting of an optical window and an ideal lens is used to simulate the imaging device.

For the imaging equipment used behind the dome window, the negative lensing effect brought by water cannot be avoided. To gain a deeper understanding of the combined impact of water and the dome window on the imaging system, we developed a simplified model using the equivalent two-optical group method to analyze alterations in system imaging. Figure 5 shows the model, wherein the entire imaging system is partitioned into two distinct components. Component 1 represents the dome window, serving to isolate the water, and component 2 represents the ideal lens, which is used in place of the imaging device. The medium behind the window is air. The radius of curvature of the front and rear surfaces of the optical window are $r_{1}$, $r_{2}$ and the thickness is $d_{l}$. The refractive index of the window material medium is $n_{g}$, and the refractive index of the object space medium is $n_{o}$ The refractive index of the image space medium is $n_{i}$.

The dome window will be used in such a way that its center of curvature coincides with the position of the entrance pupil of the subsequent imaging device. This approach offers the benefit of maintaining the unaltered object space field of view of the imaging device while effectively eliminating the dome’s coma, astigmatism, and lateral chromatic aberration, thereby minimizing their impact on image quality.

The entrance pupil is conceptually defined as the image formed by the aperture diaphragm as observed through its front element. The concentric attributes of the dome window introduce a noteworthy characteristic: when the entrance pupil of component 2 aligns with the center of curvature of the dome, the entrance pupil of the entire system co-aligns at this central point. Remarkably, this position remains fixed, remaining impervious to alterations in the object space medium, with only its size subject to modification:

(7) $$D_{w}=\frac{n_{a}}{n_{w}}D_{A}$$

where $D_{w}$, $D_{A}$ represent the system’s entrance pupil size in water and air, respectively. And $n_{a}$, $n_{w}$ represent the refractive index of air and water. In underwater conditions, the entrance pupil diameter of the underwater environmental system is reduced to $1/n_{w}$ of its original size. This reduction is one of the contributing factors to the blurring of high-frequency details in underwater images and the subsequent decrease in illuminance.

### 3.1. Underwater Optical Imaging System Focal Length

Drawing upon the calculations presented in the preceding section, the negative lens effect induced by the water window results in increased light diffusion as it traverses the optical elements. In the case of a single lens, this effect manifests as a reduction in the negative lens focal length and an increase in the positive lens focal length. Nonetheless, given that the optical system comprises multiple components, the alteration in focal length no longer adheres to the same pattern observed in a single lens configuration. The system we have modeled comprises two key components: the water primarily impacts the window component in direct contact, while the subsequent imaging component maintains its imaging properties as it consistently operates in an air medium. The alteration in the imaging properties of the entire system occurs due to changes in the focal length of the water-contacting window and the displacement of its principal plane.

**Figure 6:**
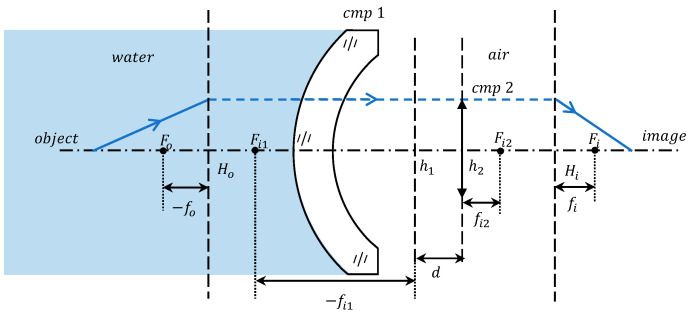
Labeling of the focal length and the principal plane for each component within the computational model (the symbols depicted in the figure exclusively indicate the direction, and the focal length extends from the principal point to the focal point).

The system’s optical power is determined using the formula applicable to a two-optical group optical system:

(8) $$\varphi =\varphi_{1}+\varphi_{2}-d\varphi_{1}\varphi_{2}$$

where φ represents the total optical power of the system, $\varphi_{1}$, $\varphi_{2}$ denote the optical power of the two components, and d stands for the optical spacing between the two components. Specifically, d is defined as the distance extending from the second principal plane of component 1 to the first principal plane of component 2, as illustrated in Figure 6. Mathematically, d is expressed as $d=h_{1}h_{2}$.

Subsequently, by utilizing the relationship between the optical power and the image-space focal length, we can determine the image-space focal length of the entire optical system, expressed as follows:

(9) $$f_{i}=\frac{n_{i}f_{i2}}{n_{i}+\varphi_{1}f_{i2}-\varphi_{1}d}$$

where $f_{i}$ is the image-space focal length of system and $f_{i2}$ is the image-space focal length of component 2. Ordinarily, the image space of the optical system is air, $n_{i}=1$. Consequently, the image-space focal length for an underwater imaging system equipped with an optical window is calculated as follows:

(10) $$f_{i}=\frac{f_{i2}}{1+\varphi_{1}\left(f_{i2}-d\right)}$$

Component 2 resides within an air medium, and its focal length, denoted as $f_{i2}$, remains unaffected by the properties of the object-space medium; it is a constant value. Consequently, the variation in the total focal length of the system is determined by both the optical power of component 1 ($\varphi_{1}$) and the optical spacing (d) between the two components. The optical power of the optical window of component 1 is the reciprocal of its image-space focal length, and the change in focal length has been calculated in the previous section. The optical spacing (d) signifies the separation between the principal planes of the two components. Component 2 is situated in an air medium, with its principal plane positions remaining constant. Therefore, to determine the change in d, it suffices to calculate the displacement of component 1’s principal planes.

**Figure 7:**
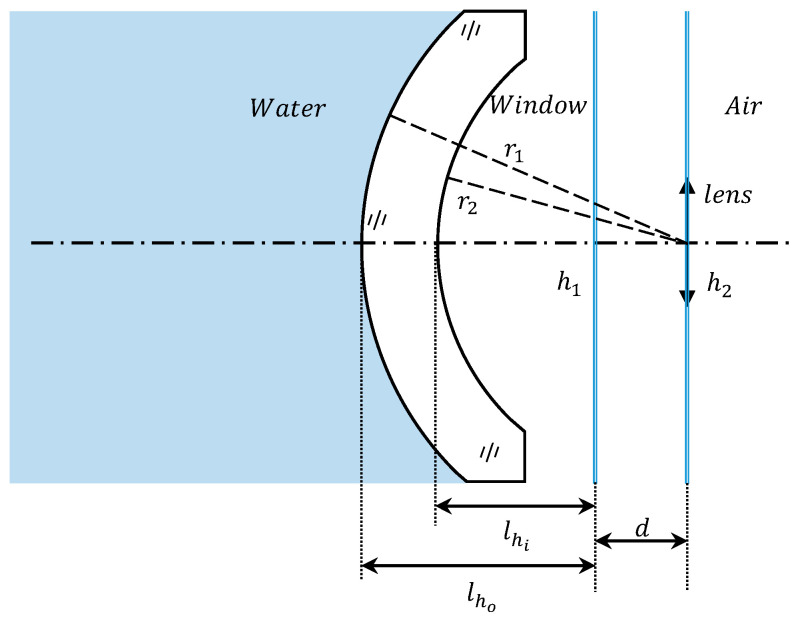
Since the first and second principal planes of the two components are each coincident, a blue line is used to represent the principal plane, and $h_{1}$, $h_{2}$ are the principal points of the two components, respectively. When transitioning from air to underwater, the principal plane of the concentric window will be shifted from the center of curvature to the direction close to the window with the increase in the refractive index of the object-space medium. This adjustment results in an expansion of the optical spacing ($d=h_{1}h_{2}$).

Component 2 is an ideal lens with both its first and second principal planes always located on its own. And component 1 features a dome window with concentric spherical surfaces, ensuring that its two principal planes perpetually align. In air, these principal planes are precisely centered at the point of curvature. When in water, the principal planes of the dome are shifted forward to more closely resemble the dome. Figure 7 provides a visual representation of the relative positions of the principal planes for both components when submerged.

According to the thick lens cardinal positions formula, the principal plane position of the dome window is obtained as follows:

(11) $$\left\{ \begin{array}{l} l_{ho}=\frac{d_{l}n_{o}\left(n_{g}-n_{i}\right)}{n_{g}r_{2}\varphi_{1}}\\ l_{hi}=\frac{d_{l}n_{i}\left(n_{o}-n_{g}\right)}{n_{g}r_{1}\varphi_{1}} \end{array}\right.$$

where the first principal plane position, $l_{ho}$, denotes the distance from the point where the front surface of the window intersects with the optical axis, serving as the starting point, to the first principal point. The second principal plane position, $l_{hi}$, represents the distance from the point of intersection between the rear surface of the window and the optical axis to the second principal point.

The optical spacing of the two components can be expressed as follows:

(12) $$d=r_{2}-l_{hi}=r_{2}-\frac{d_{l}n_{i}\left(n_{o}-n_{g}\right)}{n_{g}r_{1}\varphi_{1}}$$

When the object space medium is water, which has a greater refractive index than air, and all other conditions remain unchanged, the optical spacing between the two components increases with the object space refractive index.

In summary, the underwater environment changes the refractive index of the object space medium, resulting in changes to both the focal length of the window and the optical spacing between the two components within the system. In the formula calculation, it is crucial to consider the sign of each quantity, with $\varphi_{1}$ being a variable with a negative value and d being a variable with a positive value. And from Equation (10), we can see that the focal length $f_{i}$ decreases with the increase in the refractive index of the object-space medium.

### 3.2. Working Distance of Underwater Optical Imaging Systems

The primary issue arising from the change in focal length is its impact on the system’s imaging position. A system calibrated in air and subsequently deployed underwater faces the risk of losing its optimal imaging plane, potentially resulting in image quality no longer meeting observational requirements. Hence, we conducted calculations to determine the underwater front working distance and assess the system’s change in focus.

**Figure 8:**
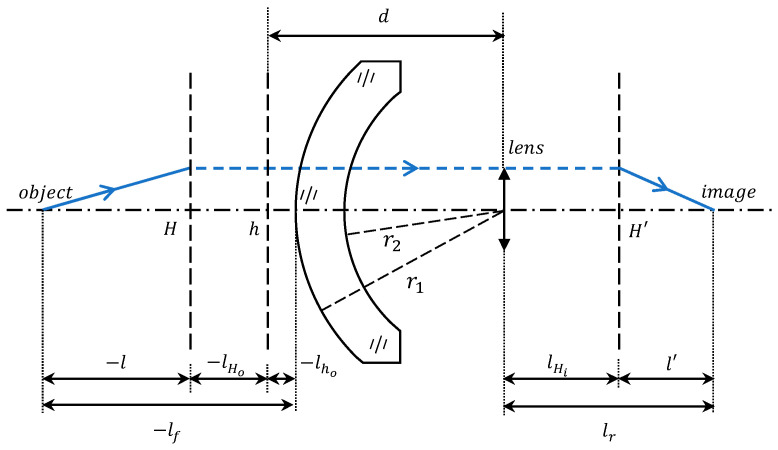
Working distance labeling for system models.

The distance at which the system can accurately focus is denoted as the front working distance $l_{f}$. It is defined as the distance from the target object that can be focused to the front vertex of the system, which corresponds to the intersection of the first face of component 1 with the optical axis. This relationship is derived from the geometric representation shown in Figure 8:

(13) $$l_{f}=l+l_{Ho}+l_{ho}$$

where $l_{Ho}$, $l_{ho}$ are the positions of the first principal planes of the system and component 1. l is the object distance, which is the distance from the second principal plane of the system to the target object. It is obtained from the geometrical optics Gaussian formula as follows:

(14) $$l=\frac{n_{o}f_{i}l^{\prime }}{f_{i}-l^{\prime }}=\frac{n_{o}f_{i}\left(l_{r}-l_{Hi}\right)}{f_{i}-l_{r}+l_{Hi}}$$

where $l^{\prime }$ represents the image distance, defined as the distance from the second principal plane of the system to the image plane. $l_{r}$ denotes the rear working distance, which signifies the distance from the rear surface of component 2 to the image plane. $l_{Hi}$ is the position of the second principal plane of the system. Therefore, the front working distance can be expressed as follows:

(15) $$\begin{array}{ll} l_{f} & =l+l_{Ho}+l_{ho}\\ & =\frac{n_{o}f_{i}\left(l_{r}-l_{Hi}\right)}{f_{i}-l_{r}+l_{Hi}}+\frac{dn_{o}}{1+\varphi_{1}\left(f_{i2}-d\right)}+\frac{d_{l}n_{o}\left(n_{g}-1\right)}{n_{g}r_{2}\varphi_{1}}\\ & =n_{o}\left[f_{i}\left(\frac{f_{i1}l_{r}+df_{i}}{f_{i}f_{i1}-l_{r}f_{i1}-df_{i}}+\frac{d}{f_{i2}}\right)+\frac{d_{l}\left(n_{g}-1\right)}{n_{g}r_{2}\varphi_{1}}\right] \end{array}$$

Furthermore, in underwater conditions, the refractive index of the medium is variable. Fluctuations in temperature, pressure, and salt content can result in the refractive index falling within a range of 1.33–1.36. The extent to which changes in refractive index affect the system’s focus needs to be confirmed.

**Figure 9:**
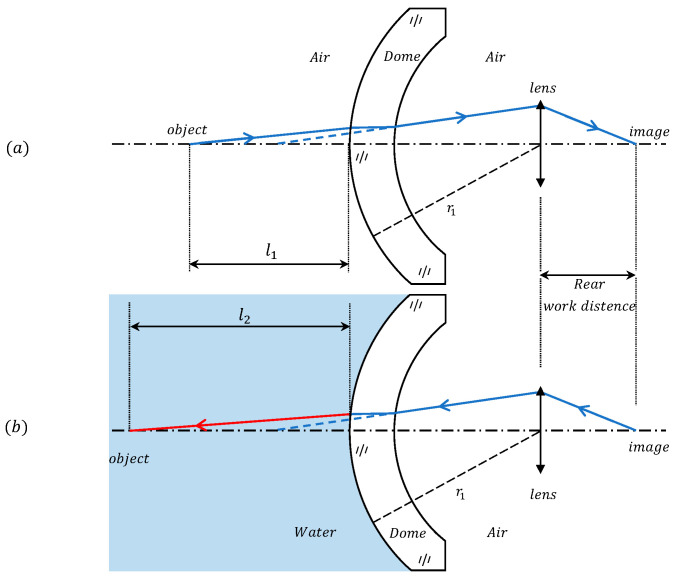
Variation of the front working distance in different media under the condition that the rear working distance is kept constant. (**a**) The light path of the system when working in air. (**b**) the Light path of the system when working underwater, utilizing the reversibility of the light path, where the light returns from the image point to the object point.

In Figure 9a, the light is emitted from the object through the imaging system and reaches the image plane, using the reversibility principle of the light path; in Figure 9b, the light returns to the object point. When the system operates in an air medium, it achieves focus on the object in position $l_{1}$. When the system’s working medium is switched to water while keeping the rear working distance constant, the front working distance extends to $l_{2}$.

It can be seen that the disparity in the light path between the two working environments solely exists at the interface of the water window. By using the refractive surface calculation formula for light [20], we can calculate the refracted light path at the water window interface as follows:

(16) $$\frac{n_{g}}{l^{\prime }}-\frac{n_{o}}{l}=\frac{n_{g}-n_{o}}{r_{1}}$$

where l, $l^{\prime }$ represent the distances from the intersection point of the light ray with the optical axis before and after refraction at the refractive surface of the water window. Since the light path remains consistent after the water window interface, i.e., $l^{\prime }$ remains constant, we can use Equation (16) to derive the relationship formula for the front working distance under varying media as follows:

(17) $$l_{2}=\frac{n_{2}}{\frac{n_{2}-n_{1}}{r_{1}}+\frac{n_{1}}{l_{1}}}$$

where $l_{1}$, $l_{2}$ are the front working distance in media 1 and 2; $n_{1}$, $n_{2}$ are refractive index in media 1 and 2; $r_{1}$ denotes the radius of curvature of the water–window interface.

Based on Equations (15) and (17), it becomes apparent that the primary factor driving alterations in the properties of the underwater imaging system is the significant variation in the refractive index of the object-space medium ($n_{o}$). And the magnitude of this change is determined by the focal length of the optical window ($f_{il}$). The change in the optical window focal length ($f_{il}$) results from the combined influence of the refractive index ($n_{o}$) and the radius of curvature of the front surface of the window ($r_{1}$). Therefore, the selection of the window shape holds significant importance for the underwater imaging system. As calculated in the preceding section, when the radius of curvature of the front surface of the window is larger, the impact of changes in the refractive index of the medium on the system is diminished.

For imaging devices, achieving a closer object focus necessitates a more distant imaging surface. Because the imaging device is encapsulated in a sealed shell, there is a limitation on the length of the fuselage, so the front working distance of the imaging device has a minimum value. When the system working environment from air to water, that is, the refractive index of the medium of the object space becomes larger, the minimum front working distance will become larger. This implies that the system’s ability to capture clear images of nearby targets is compromised when operating in high-refractive-index water environments.

### 3.3. Simulation Software Verification

In order to verify the accuracy of the calculation formula, we used the optical design software Zemax OpticStudio 19.4 [21] to simulate the paraxial imaging results of the underwater imaging system and compare them with the formula calculation results.

#### 3.3.1. Test on the Ideal System

We first simulated the ideal case by modeling the domed window and thin lens in the simulation software (Zemax OpticStudio 19.4). The optical window material in the model is sapphire, the field of view is the near-axis region, and the model parameters are set for three different cases, as shown in Table 1.

**Figure 10:**
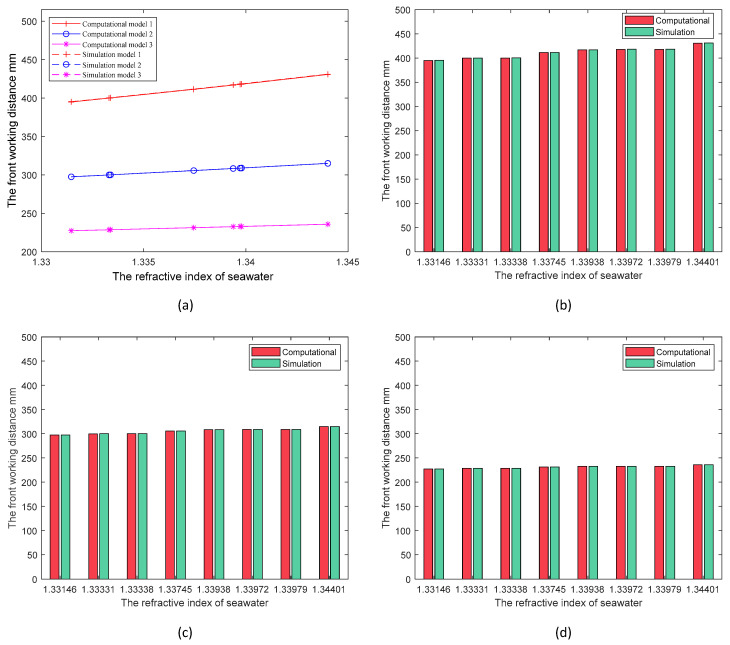
(**a**) Computational and simulation results of three models in seawater with multiple refractive indices. Data from different models are distinguished by different colors and line shapes. The solid line represents the calculation results, and the dashed line represents the simulation results; (**b**) comparison of calculation and simulation for model 1; (**c**) comparison of calculation and simulation for model 2; (**d**) comparison of calculation and simulation for model 3.

Firstly, the model working environment is set to air, with the system imaging the object at 0.1 m. After the software gives the optimal image position, other quantities are kept constant, and then the environment is changed to seawater, and the object distance is optimized to get the optimal working distance of the model in the underwater environment. We conducted simulations for eight seawater cases, varying the refractive index between 1.33 and 1.36 [16,18]. For each model, we used Equation (15) for the calculations. The final results of the calculations and simulations for all models are shown in Figure 10, where different line shapes are used to distinguish the data for different models, and solid and dashed lines are used to distinguish the calculation from the simulation results. Figure 10b–d present specific data on the front working distance obtained through both methods, corresponding to different models. The data obtained by both methods are consistent, affirming the reliability of the work distance calculation method and the accuracy of Equation (15).

The findings depicted in Figure 10 indicate a substantial increase in the system’s front working distance, expanding multiple times from 0.1 m when transitioning from air to seawater, attributable to water’s negative lensing effect on the imaging system. Additionally, in Figure 10a, the inclination of the lines also suggests that discrepancies in refractive indices within seawater contribute to alterations in the system’s front working distance. These variations may prompt a shift in the imaging system’s focusing position, leading to compromised imaging outcomes, particularly with cameras employing fixed-focus lenses. Hence, it becomes imperative to calculate the extent of change in the system’s front working distance within an underwater environment. The comparison of data among various models in Figure 10 demonstrates that the extent of variation in the front working distance correlates with the radius of curvature of the dome surface, wherein cameras fitted with larger-radius domes exhibit diminished variations in the front working distance across diverse environments, displaying enhanced adaptability. Opting for an appropriate dome window shape proves beneficial for cameras operating in specific underwater environments, as it mitigates the impact of focus point shifts on imaging.

#### 3.3.2. Test on the Real System

**Figure 11:**
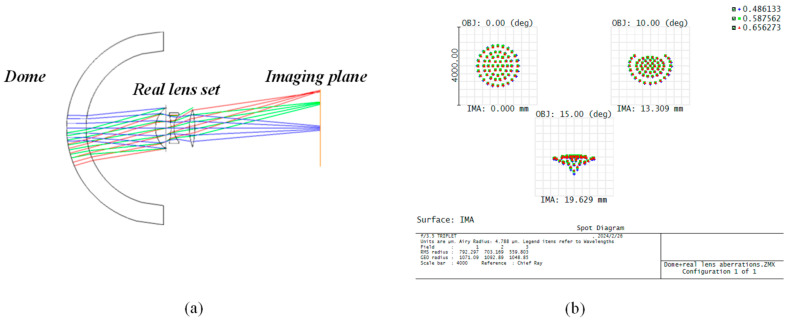
Using the real lens, there are large aberrations, and the imaging quality is very poor. (**a**) The layout of the imaging system using the real lens set; (**b**) the dot plot of the imaging system using the real lens set. The light spot is highly dispersed, exhibiting significant aberrations.

To further validate the accuracy of our computational method, we incorporated real lenses behind the dome for simulation. In Figure 11a, we employed the objective lens from the simplest Cooke three-piece set [22] as the real lens configuration behind the dome. The optical parameters are specified in Table 2. Initially, we consider the actual imaging conditions where the imaging area extends beyond the near-axis range to encompass the entire aperture of the imaging system and the off-axis field of view. This expansion enlarges the lateral range of the entire imaging system, resulting in significant aberrations. Under conditions of large aperture and wide field of view, even with an optimized lens configuration, the combination of the dome and water induces unavoidable aberrations, leading to diminished image quality, as illustrated in Figure 11b.

**Figure 12:**
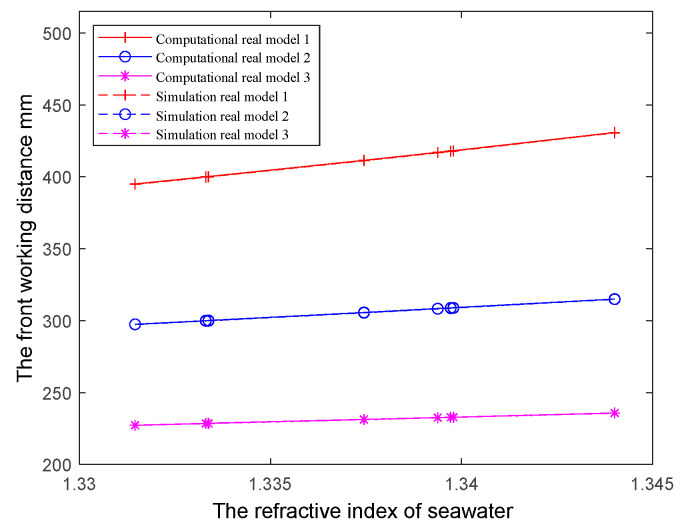
Computational and simulation results of three real models in seawater with multiple refractive indices.

To minimize the effect of aberrations, when using real lenses, the entire system can be approximated as ideal imaging if the conditions are limited to the near-axis case. We employed the three models listed in Table 3, where the dome data remained consistent with the ideal lens scenario but with the use of a real lens group following the spherical shell. The calculation results derived from the formulas and those from Zemax simulations are illustrated in Figure 12. Both approaches demonstrate the same change pattern, with very closely matched data results. While the real lens simulation is based on conditions of near-axis optical imaging, the results further validate the accuracy of our derived formulas for the front working distance and other parameters. This significantly aids in optimizing the calculation of underwater imaging systems and provides valuable guidance for constructing and designing a reasonable and feasible imaging system. In the next step, we will solve the problem of the aberration between the dome window and the water and further realize the qualified imaging quality in the lateral range of the imaging system behind the dome.

### 3.4. Depth of Field for Underwater Imaging Systems

In practical optical systems, the specific object position corresponding to a clear image is no longer fixed, allowing for a certain degree of variation in the object distance. Objects within the depth of field range can fulfill the conditions required for clear imaging. When the object space environment changes, we need to redefine the depth of field range of the imaging system. When transitioning from an air to an underwater environment, if the change in system focus distance falls within the depth of field, the system initially calibrated on land can meet the requirements for underwater use and simplify the focusing process when operating underwater. To solve this problem, we derive a depth-of-field formula applicable to underwater imaging systems.

Most books on geometrical optics give a formula for the system depth of field [23]:

(18) $$\Delta =\frac{-2\delta Df_{i}l\left(l+f_{i}\right)}{\delta^{2}{\left(l+f_{i}\right)}^{2}-D^{2}{f_{i}}^{2}}$$

where Δ is the depth of field of the system; δ is the maximum permissible dispersion circle diameter; D is the Entrance pupil diameter; l is focus distance; $f_{i}$ is the focal length in the image space of the system.

**Figure 13:**
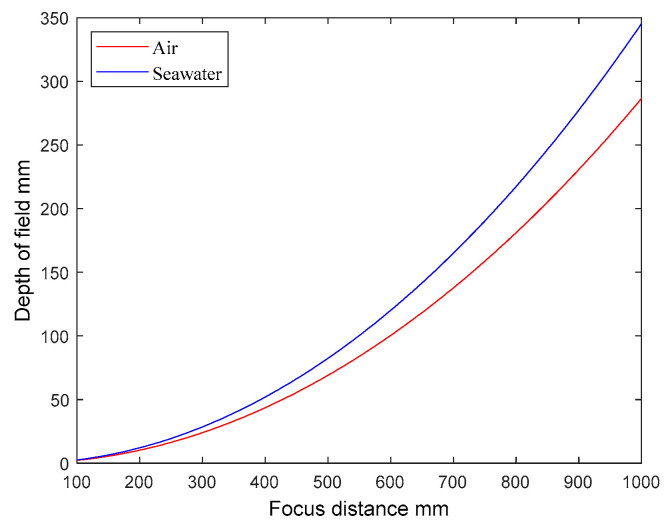
Curve of the depth of field versus focusing distance for the same optical system when in air and seawater, respectively.

The formula above applies to systems where the object space is air. As the refractive index of the object space medium is a significant factor influencing the depth of field, a modification is required when the object space medium changes from air to water. The adjusted depth of field formula becomes the following:

(19) $$\Delta_{n}=\frac{-2\delta Df_{i}l\left(l+n_{o}f_{i}\right)}{\delta^{2}{\left(l+n_{o}f_{i}\right)}^{2}-D^{2}{n_{o}}^{2}{f_{i}}^{2}}$$

where $\Delta_{n}$ is the new depth of field of the system; $n_{o}$ is the refractive index of the object space medium. From Equation (19), multiple factors exert influence over the depth of field. The focal length, entrance pupil diameter, and focusing distance collectively determine the extent of the depth of field. Additionally, changes in the refractive index of the water introduce variations in the depth of field range. As an illustrative calculation, we selected the system parameters outlined in Section 3.3. And the diameter of the incident pupil is 10 mm, the maximum permissible dispersion circle diameter is 30 μm, the focusing distance is 100–1000 mm and uses Equation (19) to calculate the depth of field ranges in air and seawater, respectively, and the results are shown in Figure 13.

According to Equation (19) and Figure 13, it is evident that the depth of field for the same imaging system expands as the refractive index of the object space medium increases at a consistent focusing distance. And the imaging system will have a greater depth of field underwater compared to in air. However, this relationship is not a straightforward n_o-fold increase, and the depth of field underwater grows much more rapidly with the increase in the focusing distance, which is much more conducive for the imaging system to be used for focusing when the imaging system is used underwater.

## 4. Conclusions

In contrast to imaging in air, the uniqueness of underwater imaging is due to how water and windows affect light transmission. This paper outlines the principles governing the performance variations of underwater imaging systems. This paper begins by treating the window as a thick lens and deducing the formula for calculating the underwater focal length of the window. It then concludes the rules governing focal length changes under two water–window shapes and identifies the characteristics of the window that is less affected by water. By establishing a geometric model of light propagation and using paraxial geometric optics calculations, the paper proceeds to derive the focal length and working distance of underwater systems and derived a method to calculate the offset in the focusing position when the camera operates underwater. It establishes the change in the refractive index of the object space as the primary influencing factor and corrects the depth-of-field formula. This correction renders the formula suitable for imaging systems operating in underwater environments. In addition, we verified the accuracy through optical design software. In the future, our work will focus on applying the working distance formula to the focusing calculation and image processing of underwater imaging systems to ensure the improvement of underwater imaging quality and to solve the aberration problem introduced by the dome window to further improve the clarity of underwater imaging.

## Figures and Tables

**Table 1:** Optical Parameters of the Zemax Model.

Optical Parameters	Model 1	Model 2	Model 3
Front curvature radius of the window	50 mm	60 mm	80 mm
Rear curvature radius of the window	40 mm	50 mm	70 mm
Axial thickness of the window lens	10 mm	10 mm	10 mm
Focal length of the ideal lens	20 mm	20 mm	20 mm

**Table 2:** Optical Parameters of the real system.

Optical Parameters	Real System
Focal length of ideal lens	50 mm
F-number	3.5
Entrance pupil position (relative to the first side of the lens)	4.103 mm
Distance between the dome and real lens	35.897 mm
Front curvature radius of the window	50 mm
Rear curvature radius of the window	40 mm

**Table 3:** Parameter settings for the three real models.

Optical Parameters	Real Model 1	Real Model 2	Real Model 3
The radius of curvature of the first surface	50 mm	60 mm	80 mm
The radius of curvature of the second surface	40 mm	50 mm	70 mm
Axial thickness of the window lens	10 mm	10 mm	10 mm
Focal length of the real lens	50 mm	50 mm	50 mm

## Data Availability

Data are contained within the article.
